# Polyphenol-Loaded Liposomal Nanocarriers from *Marrubium vulgare*: A Promising Nutraceutical Delivery System with Enhanced Bioactivity and Safety

**DOI:** 10.3390/molecules31152660

**Published:** 2026-07-30

**Authors:** Youssra Lefrioui, Fabrizia Sepe, Raffaele Conte, Anna Calarco, Wessal Ouedrhiri, Mohamed Chebaibi, Ahmad Mohammad Salamatullah, Razan M. Salamatullah, Mohammed Bourhia, Musa A. Said, Andriy Grafov, Dalila Bousta

**Affiliations:** 1 Laboratory of Biotechnology Environment Agrofood and Health (LBEAS), Faculty of Sciences Dhar El Mahraz, Sidi Mohamed Ben Abdellah University, Fez 30000, Morocco; lefyoussra@gmail.com (Y.L.); dalila.bousta@usmba.ac.ma (D.B.); 2 Research Institute on Terrestrial Ecosystems (IRET)—CNR, 80131 Naples, Italy; fabriziasepe@cnr.it; 3 Algal Biotechnology Centre, University Mohamed 6 Polytechnic (UM6P), BenGuerir 43150, Morocco; wessal.ouedrhiri@um6p.ma; 4 Ministry of Health and Social Protection, Higher Institute of Nursing Professions and Health Techniques, Fez 30050, Morocco; mohamed.chebaib@usmba.ac.ma; 5 Department of Food Science & Nutrition, College of Food and Agricultural Sciences, King Saud University, 11 P.O. Box 2460, Riyadh 11451, Saudi Arabia; asalamh@ksu.edu.sa; 6 Department of Pharmaceutical Sciences, Faculty of Pharmacy, Umm Al-Qura University, Makkah 21955, Saudi Arabia; s442007142@uqu.edu.sa; 7 Laboratory of Biotechnology and Natural Resources Valorization, Faculty of Sciences, Ibn Zohr University, Agadir 80060, Morocco; m.bourhia@uiz.ac.ma; 8 Department of Chemistry, Faculty of Science, Islamic University of Madinah, Madinah 42351, Saudi Arabia; mssaeed@iu.edu.sa; 9 Department of Chemistry, Faculty of Sciences, Helsinki University, 00100 Helsinki, Finland; andriy.grafov@helsinki.fi; 10 National Agency of Medicinal and Aromatic Plants, Taounate 34025, Morocco

**Keywords:** *M. vulgare*, polyphenolic extract, liposomes, nanoformulation, bioavailability, anti-inflammatory activity, analgesic effect, drug delivery system

## Abstract

*Marrubium vulgare* L. aerial parts are a rich source of polyphenols with recognized antioxidant and anti-inflammatory properties; however, their therapeutic potential is limited due to poor stability and bioavailability. To enhance its pharmacological efficacy, a liposomal formulation of *M. vulgare* polyphenolic extract (MV-Lipos) was developed in this study by employing the thin-film hydration method. Before encapsulating, the free-extract (MV-Ext) was analyzed using LC-MS, and the resultant nanoliposomes were tested for physicochemical qualities, biological activity, and safety. MV-Lipos exhibited particle sizes ranging from 127 to 200 nm, an 84% encapsulation efficiency, and high colloidal stability (zeta potential −29.58 ± 0.40 mV). In vitro evaluations revealed anti-inflammatory and antioxidant activities without cytotoxic effects. In vivo, MV-Lipos significantly improved analgesic and anti-inflammatory responses. Specifically, a dose of 100 mg/kg lowered acetic acid induced writhing by up to 75.9% and carrageenan induced paw edema by 72%, with efficacy comparable to ibuprofen. A 28-day subacute toxicity assessment found no treatment-related adverse effects. Furthermore, molecular docking analyses validated the experimental results by revealing possible interactions with inflammation-related targets. Overall, liposomal encapsulation improved the biological efficacy and safety profile of *M. vulgare* polyphenols, highlighting their potential as natural agents for the management of pain and inflammatory conditions.

## 1. Introduction

Plants are a rich source of bioactive compounds with diverse pharmacological activities, many of which are attributed to secondary metabolites such as polyphenols, alkaloids, and terpenoids [1]. Among these species, *Marrubium vulgare* L., (white horehound, Lamiaceae) has long been recognized in traditional medicine for its therapeutic properties, particularly in the treatment of respiratory disorders, inflammatory conditions, and pain-related ailments [2,3,4].

**Scheme 1:**
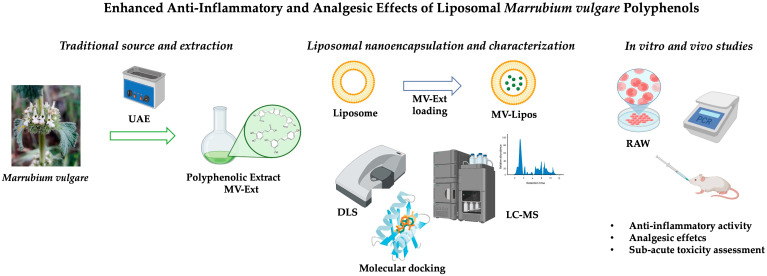
Schematic representation of the study workflow and research methodology.

The pharmacological effects of *M. vulgare* are mainly associated with its complex phytochemical profile, which includes diterpenoids (e.g., marrubiin), phenylpropanoids, and polyphenols [5]. Several studies have reported its antioxidant, antimicrobial, hypoglycemic, analgesic, and anti-inflammatory activities [6,7,8]. Among its constituents, plant-derived polyphenols play a key role in modulating inflammatory responses through the inhibition of key signaling pathways such as MAPK, AP-1, and NF-κB [9,10]. However, despite their promising biological potential, the clinical translation of polyphenols is often limited by their poor aqueous solubility, chemical instability, and low bioavailability resulting from degradation in the gastrointestinal tract and rapid metabolism [11,12,13]. To address these challenges, the use of nanocarrier-based delivery systems has gained increasing attention to improve the pharmacokinetic and pharmacodynamic properties of natural bioactive compounds [14]. Among them, liposomes have emerged as one of the most versatile and biocompatible systems due to their structural similarity to biological membranes, their ability to encapsulate both hydrophilic and hydrophobic molecules, to improve compound stability, to provide controlled release, and to potentially enhance bioavailability [15]. These characteristics broaden the application of liposomes to the delivery of antibiotics, anticancer agents, anti-inflammatory drugs, antifungal medications, anesthetics, and a plethora of other pharmaceutical substances [16,17,18,19,20]. Recently, liposome encapsulation has been successfully applied to several natural compounds, such as curcumin [21], resveratrol [22], quercetin [12], and caffeic acid [23], improving their pharmacokinetic profiles and therapeutic efficacy [24]. Building on this background, the present study aimed to develop and characterize a liposomal formulation of the polyphenolic extract from *M. vulgare* (MV-Lipos). The formulation was evaluated for its physicochemical properties, including particle size, zeta potential, and encapsulation efficiency, as well as for its stability and safety. Furthermore, the in vivo pharmacological activities—analgesic and anti-inflammatory effects—were assessed in murine models, along with a subacute toxicity study to ensure safety. In silico molecular docking was also performed to support and elucidate possible mechanisms of action at the molecular level. While phospholipid-based liposomes represent a well-established delivery technology, this study constitutes the first comprehensive approach to encapsulate and validate this specific polyphenolic fraction, bridging the gap between nano-formulation design and multi-target biological evaluation. Overall, this work seeks to provide new insights into the development of biocompatible nanoformulations of plant-derived polyphenols. Ultimately, it aims to demonstrate the potential of liposomal encapsulation as a promising strategy for enhancing the therapeutic efficacy of *M. vulgare* in the management of inflammation and pain (Scheme 1).

## 2. Results

### 2.1. Qualitative Profiling of the M. vulgare Phenolic Compounds by LC/MS Total Phenol Content

**Figure 1:**
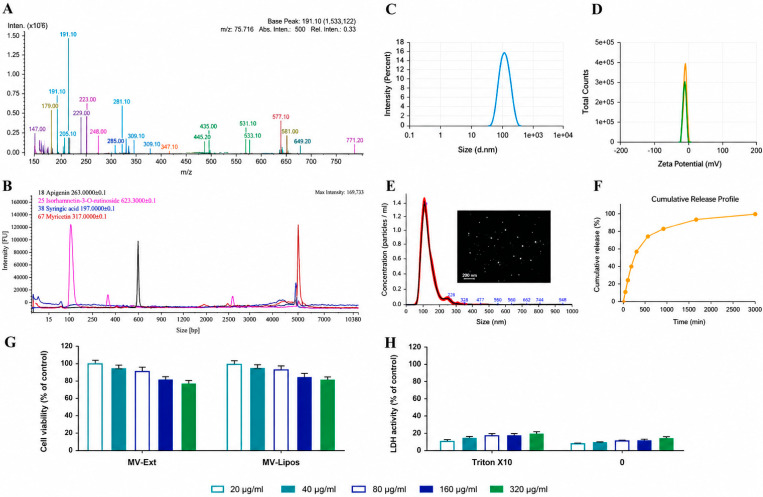
(**A**,**B**) LC-MS Profile of *Marrubium vulgare* Polyphenol Extract with Peak Identification in Table 1. (**C**–**E**) Physicochemical characterization of polyphenol-loaded liposomes (MV-Lipos). (**C**) Size distribution histogram obtained by dynamic light scattering (DLS). (**D**) Zeta potential distribution. (**E**) Nanoparticle tracking analysis (NTA) profile with Representative camera snapshot from the NTA acquisition, visualizing individual liposomes in Brownian motion. (**F**) Cumulative MV release. (**G**) Cell viability and (**H**) LDH release of RAW cells after incubation with MV-Ext and various concentrations of liposome formulations for 24 h.

The present study aimed to formulate polyphenol-loaded liposomes using *M. vulgare* extracts to improve bioavailability and efficacy. In this regard, both the free extract (MV-Ext) and the polyphenol-loaded liposomal formulations (MV-Lipos) were subjected to pharmacological evaluation. The analysis focused on their analgesic and anti-inflammatory activities, as well as sub-acute toxicity assessments to determine their safety profile. To support the formulation strategy, the polyphenolic composition of MV-Ext was characterized through LC-MS/MS analysis. The qualitative and quantitative profiling of the polyphenolic fraction revealed several phenolic compounds with varying concentrations, as illustrated in (Figure 1A,B) and detailed in Table 1. In particular, the total phenolic content of MV-Ext, measured using the Folin–Ciocalteu reagent, showed a content of 126.51 ± 0.002 mg GAE per g for the crude *M. vulgare* extract. Among the most abundant constituents, syringic acid (358.8 mg/100 g), verbascoside (238.6 mg/100 g), and rutin (79.25 mg/100 g) were predominant. Other notable compounds detected included resveratrol (25 mg/100 g), isorhamnetin-3-O-rutinoside (25.04 mg/100 g), myricetin (7.92 mg/100 g), naringin (4.37 mg/100 g), procyanidin (10.61 mg/100 g), kaempferol-3-O-glucuronic acid (1.58 mg/100 g), luteolin-7-O-glucoside (1.31 mg/100 g), sinapic acid (1.82 mg/100 g), ferulic acid (1.26 mg/100 g), apigenin (3.42 mg/100 g), and catechin/epicatechin (1.61 mg/100 g). Compounds such as quercetin-3-O-glucoside and isorhamnetin-7-O-pentose were also identified, although in minor amounts (<0.1 mg/100 g). These results emphasize the complex and rich polyphenolic profile of MV-Ext, which underpins its potential biological activity.

### 2.2. Liposome Fabrication, Characterization, and In Vitro Cytotoxicity

Liposomes have been proven to be effective carriers for both lipophilic and hydrophilic molecules and to enable sustained and regulated release of entrapped compounds providing several benefits in localized treatment [25]. Their effectiveness as a delivery method can be maximized by adjusting their size, polydispersity, and surface properties. In this study, the well-known thin-film hydration (TFH) technique was used for the preparation of MV-Lipos [26]. To our best knowledge, this is the first example of a liposomal formulation for *M. vulgare* polyphenols. MV-Lipos was physiochemically characterized by Z-average diameter, polydispersity index (PDI), and zeta potential. As shown in Figure 1C, the nanocarriers exhibited a hydrodynamic diameter of approximately 127.5 ± 1.5 nm, as determined by dynamic light scattering (DLS), remaining well within the optimal size range (<200 nm) for biomedical applications. The polydispersity index (PDI) was recorded at 0.26 which indicates a narrow size distribution and a moderate formulation homogeneity. The zeta potential was found to be –29.58 ± 0.40 mV, indicating a moderately high negative surface charge (Figure 1D). Additionally, nanoparticle tracking analysis (NTA) revealed a particle concentration of (8.38 ± 0.14) × 10^8^ particles/mL, confirming a high density of nanocarriers in suspension (Figure 1E). The consistency between NTA and DLS data supports the robustness of the preparation method.

Moreover, the physical stability of the MV-Lipos formulation was evaluated by monitoring particle size and zeta potential during storage in PBS buffer over a period of 21 days. After 21 days of storage, only a limited variation in these parameters was observed, with particle size increasing by approximately 3% to 131.3 ± 1.5 nm, while the zeta potential showed a variation of only 1.2%, reaching −29.23 ± 0.40 mV. The negligible changes in particle size suggest that the liposomal vesicles retained their colloidal stability without significant aggregation or fusion phenomena during storage. Similarly, the preservation of the negative surface charge indicates that the electrostatic properties of the phospholipid bilayer remained substantially unchanged, contributing to the maintenance of vesicle stability.

Then, to further elucidate the release mechanism of MV-Lipos, the experimental release data were analysed according to the Korsmeyer–Peppas kinetic model. The model showed the best fitting performance among the tested kinetic approaches, with a high correlation coefficient (R^2^ ≈ 0.92) calculated over the initial phase of release. The release exponent (*n*), calculated from the relationship between the fraction of polyphenols released (Mt/M∞) and time, was found to be approximately 0.6, indicating an anomalous transport mechanism. The release profile of polyphenols from MV-Lipos, shown in Figure 1F, exhibited a typical biphasic behaviour, characterised by an initial burst phase followed by a prolonged sustained release phase. Approximately 21.5% of the MV-Lipos were released within the first hour, whereas the cumulative release progressively increased to 75.5 ± 3.1% after 72 h.

The encapsulation efficiency (EE) of MV-Lipos was 84% (0.042 ± 0.01 mg GAE/mL) with respect to the measured content in the encapsulated extract (at 0.05 mg/mL), as determined by LC-MS/MS detection. In particular, the obtained results showed high encapsulation efficiencies for all analysed compounds, with values of 84% for syringic acid, 81% for verbascoside, and 87% for quercetin. Despite their different polarity profiles, all selected compounds exhibited efficient incorporation into the liposomal system. Verbascoside, characterized by a higher hydrophilic character due to the presence of glycosyl residues, showed a slightly lower encapsulation efficiency (81%), which may be attributed to its preferential localization within the aqueous phase of the liposomes and possible steric limitations associated with its larger molecular structure. Conversely, quercetin, a less polar flavonoid aglycone, showed the highest encapsulation efficiency (87%), likely due to stronger hydrophobic interactions with the phospholipid bilayer. Syringic acid, representing a smaller and more polar phenolic acid, also exhibited efficient encapsulation (84%), suggesting that both hydrogen bonding interactions and partitioning within the interfacial region of the lipid membrane may contribute to its retention.

The biocompatibility of nanomaterials plays a critical role in maintaining cell viability and proliferation. To further assess the compatibility of the liposomes, RAW264.7 macrophages were cultured in presence of varying concentrations of MV-Lipos. As shown in (Figure 1G), no significant decrease in cell viability was observed in either RAW264.7, with cell viability equal to or higher than 80% compared with the untreated control group, even at concentrations up to 200 μg/mL. Similar results were obtained with the measurement of released LDH from cells (Figure 1H). MV-Lipos did not induce any significant increase in LDH activity compared to the non-treated cells, confirming no significant toxicity at any of the tested concentrations. Based on these data, 100 μg/mL was selected as the optimal working concentration for subsequent in vitro experiments.

This delivery system represents a promising strategy that may contribute to improve the oral delivery, slowing down their breakdown, and lowering the toxicity of polyphenols. However, future pharmacokinetic studies are required to definitively confirm its effect on bioavailability.

### 2.3. MV-Lipos Induces ROS Scavenging and Inflammation Attenuation in LPS-Stimulated RAW264.7 Macrophages

**Figure 2:**
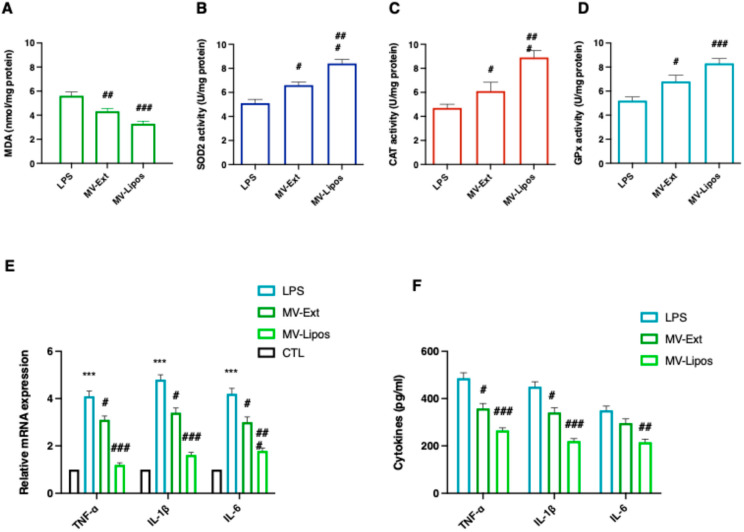
The effect of the MV polyphenols on LPS-induced macrophages. (**A**–**D**) ROS-scavenging ability of MV-Ext and MV-Lipos in RAW macrophages. (**A**) MDA level, (**B**) superoxide dismutase (SOD2), (**C**) catalase (CAT), and (**D**) glutathione peroxidase (GPx) activities were measured using the commercial assay kit. (**E**) Relative mRNA expression of *TNF-α*, *IL-1β*, and *IL-6* determined by RT-qPCR after LPS stimulation (100 μg/mL) of RAW264.7 cells for 24 h and then incubated in presence of MV-Ext or MV-Lipos. Stimulated macrophages were compared with unstimulated cells used as control (CTL). Data are represented as fold change over actin (2^−ΔΔCt^). (**F**) ELISA quantification of pro-inflammatory (TNF-α, IL-1β, and IL-6) mediators in LPS-stimulated macrophages. Results are expressed as the means of three independent experiments ± SD. (n = 3). Statistically significant variations: *** *p <* 0.001 versus control; # *p* < 0.05, ## *p* < 0.01, and ### *p* < 0.001 versus LPS.

Reactive oxygen species (ROSs) are produced because of oxidative metabolism under physiological conditions and are counterbalanced by scavenging and generation processes [27]. ROSs are essential for several biological processes, including cell survival, proliferation, and even immune response in both normal and pathological conditions. However, during inflammatory conditions, macrophages (MΦs) increase the production of a ROS as a defense mechanism that disturbs homeostasis and predisposes macrophages to the M1 pro-inflammatory phenotype, leading to various diseases such as atherosclerosis, diabetes, rheumatoid arthritis, and inflammatory bowel disease (IBD) [28]. Therefore, scavenging of ROSs represents an effective antioxidative strategy to treat many inflammatory diseases. In this study, an inflammatory microenvironment model was constructed using LPS to explore the anti-inflammatory and immunomodulatory effects of MV-Lipos. As expected, treatment of MΦ cells with LPS significantly increased intracellular ROS generation. Concurrently, a marked decrease in the activities of superoxide dismutase (SOD), catalase (CAT), and glutathione peroxidase (GSH-Px) was observed compared to untreated controls (Figure 2B–D). Treatment with MV-Ext attenuated this ROS overproduction recovering the activity of antioxidant enzymes. Notably, treatment with MV-Lipos led to a significantly stronger enhancement (*p* < 0.01) of the antioxidant enzymatic defense system. Accordingly, the presence of MV-Lipos resulted in a significant (*p* < 0.01) reduction in intracellular lipid peroxidation, as indicated by decreased malondialdehyde (MDA) production (Figure 2A).

Increased oxidative stress has been identified as main source of chronic inflammation. Phenolic antioxidants modulate the activities of lymphocytes and macrophages by impacting cytokine and nitric oxide release. They exert anti-inflammatory effects by counteracting the excessive secretion of pro-inflammatory cytokines such as IL-1β, IL-6, and IL-18, thereby mitigating chronic inflammation. RT-qPCR analysis was conducted to monitor the transcriptional expression of key inflammatory mediators following treatment. As shown in Figure 2E, the expression of pro-inflammatory genes including IL-1β, IL-6, and TNF-α, was significantly increased in LPS group (*p* < 0.05). On the contrary, the presence of MV-Lipos at a concentration of 100 µg/mL induces a significant (*p* < 0.001) downregulation of mRNA expression of these pro-inflammatory mediators, whereas MV-Ext exhibited only modest effects (*p* < 0.05).

Consistently, ELISA results corroborated the RT-qPCR data. Treatment with MV-Lipos significantly (*p* < 0.01) reduced the release of IL-1β, IL-6, and TNF-α cytokines into the microenvironment in activated macrophages compared to LPS-activated controls. Notably, while MV-Ext also reduced the levels of these cytokines, the effect of MV-Lipos was markedly greater (Figure 2F). This suggests that liposomal encapsulation helps the extract to mediate anti-inflammatory effects by inhibiting pro-inflammation cytokines. However, future studies are needed to confirm the precise molecular mechanisms underlying this inhibition, as key intracellular signaling cascades like NF-kB and MAPK were not directly investigated.

### 2.4. Assessment of the Toxicological Impact of M. vulgare Extracts

**Figure 3:**
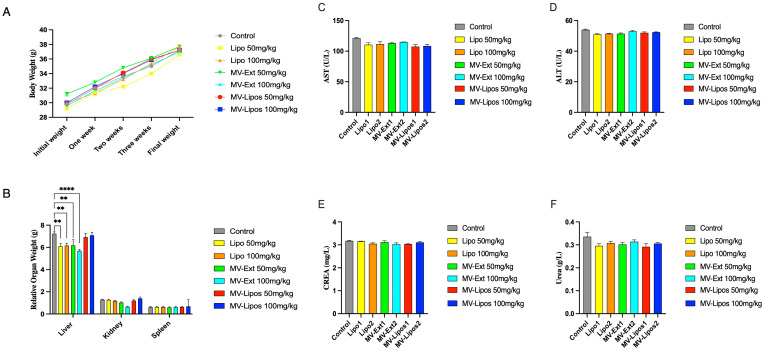
Sub-acute toxicity evaluation of Lipo, MV-Ext and MV-Lipos in mice over a 3-week treatment period. (**A**) Body weight progression monitored weekly from initial allocation to final weight. (**B**) Relative organ weights (liver, kidney, and spleen) determined at the end of the study. Serum biochemical quantification of liver function markers: (**C**) aspartate transaminase (AST) and (**D**) alanine aminotransferase (ALT). Kidney function indicators: (**E**) creatinine (CREA) and (**F**) urea. Data are expressed as Mean ± SEM (n=5 per group). Significant differences are indicated as **p<0.01 and ****p<0.0001, while non-marked comparisons represent non-significant variations (p>0.05).

The toxicity of *M. vulgare* polyphenolic extracts was evaluated to assess the potential toxic effects of both encapsulated and non-encapsulated polyphenolic extracts. All mice exhibited weight gain over the 28-day treatment period (Table 2, Figure 3A). No signs of toxicity were observed, including fever, allergic reactions, weakness, redness, or disorders affecting the digestive, nervous, or muscular systems. The relative vital organ weights (ROWs) for the kidney, liver, and spleen are provided in Table 3 and illustrated in Figure 3B. After 28 days of oral administration of MV-Lipos, MV-Ext and blank liposomes (Lipo) at doses of 50 and 100 mg/kg, the relative weights of the kidneys and spleen remained within the range of the control group. Interestingly, a statistically significant decrease in relative liver weight was observed in mice treated with Lipo and MV-Ext compared to the control. Conversely, mice orally treated with MV-Lipos maintained normal liver weights, showing no statistically significant differences compared to the untreated control group, thus indicating a protective effect of liposomal encapsulation.

The biochemical parameters of the treated and control animals, including aminotransferases (AST and ALT), urea, and creatinine, are presented in Table 4 and in Figure 3C–F. The results indicate that repeated oral administration of MV-Ext and MV-Lipos, at a maximum dose of 100 mg/kg per day for 4 weeks, showed no significant changes in plasma levels of urea, creatinine, or liver enzyme markers (ALT and AST).

**Figure 4:**
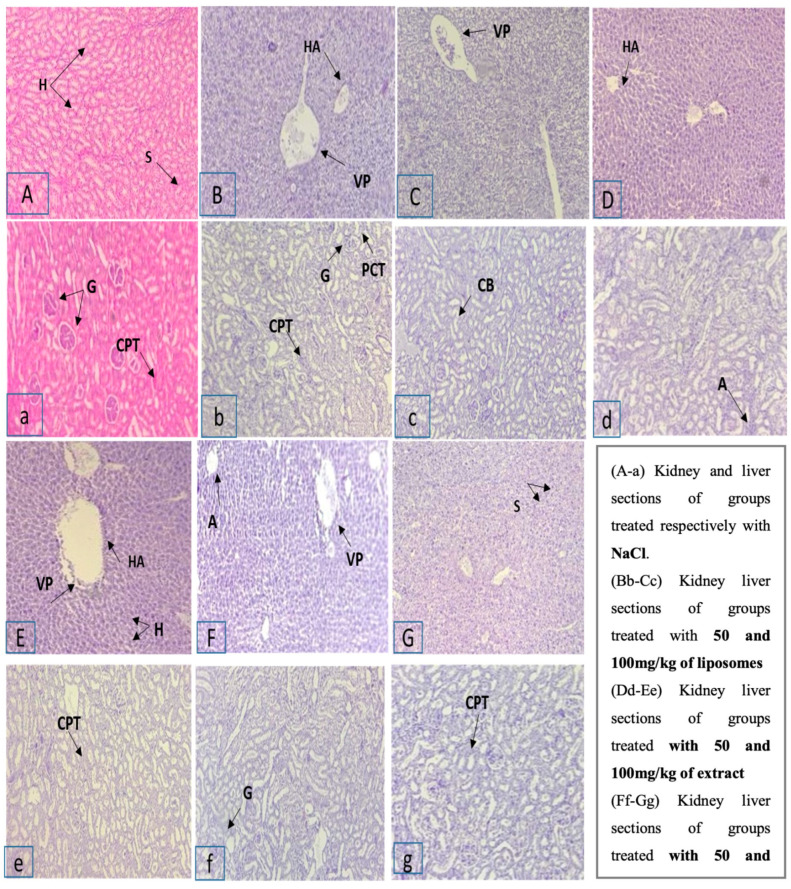
Histopathological microscopies of kidney and liver sections stained with hematoxylin–eosin after oral application of *M. vulgare* MV-Ext and MV-Lipos in mice on day 28 of subacute toxicity. (**A**) liver section of NaCl control group; (**a**) kidney section of NaCl control group; (**B**) liver section of 50 mg/kg Lipo group; (**b**) kidney section of 50 mg/kg Lipo group; (**C**) liver section of 100 mg/kg Lipo group; (**c**) kidney section of 100 mg/kg Lipo group; (**D**) liver section of 50 mg/kg MV-Ext group; (**d**) kidney section of 50 mg/kg MV-Ext group; (**E**) liver section of 100 mg/kg MV-Ext group; (**e**) kidney section of 100 mg/kg MV-Ext group; (**F**) liver section 50 mg/kg of MV-Lipos group; (**f**) kidney section 50 mg/kg of MV-Lipos group; (**G**) liver section 100 mg/kg of MV-Lipos group; (**g**) kidney section 100 mg/kg of MV-Lipos group. Abbreviations: H: hepatocytes; S: sinusoids; HA: hepatic artery; VP: portal vein; G: glomerulus; CPT: peritubular capillaries; PCT: proximal convoluted tubule; A: arteriole; CB: bile canal.

Histopathological examination of the kidneys and livers from treated mice, in comparison to the control group, revealed no lesions or pathological changes attributable to the pre-treatment. Microscopic analysis of liver and kidney sections from animals receiving MV-Ext and MV-Lipos (at doses of 50 and 100 mg/kg) showed no significant pathological differences compared to the control group (Figure 4).

### 2.5. Analgesic and Anti-Inflammatory Activities

**Figure 5:**
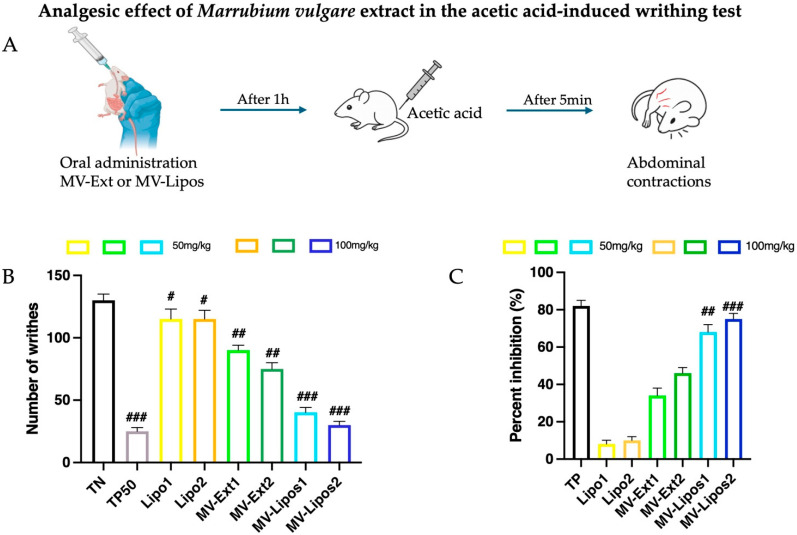
Analgesic activity of encapsulated and non-encapsulated polyphenolic extract of *M. vulgare* on acetic acid-induced writhing reflex in mice. (**A**) Schematic representation of the in vivo experimental protocol. (**B**) Number of abdominal writhes recorded in mice treated with vehicle (TN), positive control (TP, 50 mg/kg), Lipo (50–100 mg/kg), MV-Ext (50–100 mg/kg), and MV-Lipos (50–100 mg/kg). (**C**) Percent inhibition of cramps in treated groups. Results are expressed as means ± SD of five independent experiments. Statistical comparisons were performed against the controls as follows: ^#^ *p* < 0.05, ^##^ *p* < 0.01, ^###^ *p* < 0.001.

The intraperitoneal injection of acetic acid induced 126.2 writhes in the negative control group (Figure 5). Meanwhile, pre-treatment with MV-Lipos at doses of 50 and 100 mg/kg, significantly reduced the number of writhes by 67.03% and 75.91%, respectively. Interestingly, the analgesic effect of MV-Lipos at a dose of 50 mg/kg was comparable to that of tramadol [29].

**Figure 6:**
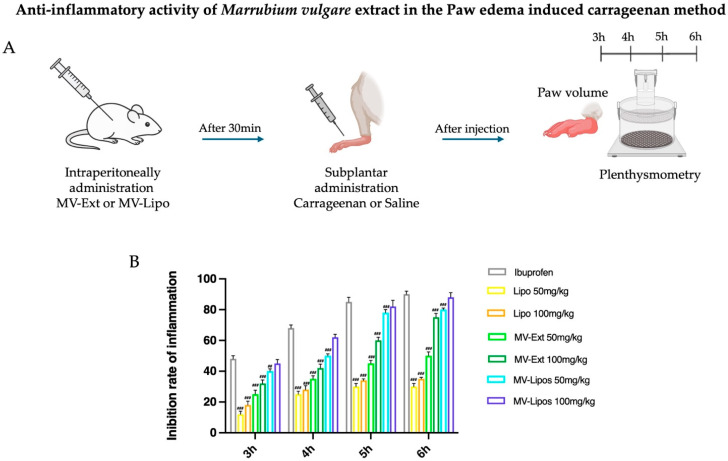
Anti-inflammatory effect of Lipo, MV-Ext, and MV-Lipos in the carrageenan-induced paw edema model. (**A**) Schematic illustration of the in vivo protocol. (**B**) Percentage of edema inhibition at 3, 4, 5, and 6 h after carrageenan injection in mice treated with ibuprofen, Lipo (50–100 mg/kg), MV-Ext (50–100 mg/kg), and MV-Lipos (50–100 mg/kg). Data are reported as mean ± SD from five independent experiments (n = 5). Significant differences in each lot versus the controls were as follows: ^#^ *p* < 0.05, ^##^ *p* < 0.01, ^###^ *p* < 0.001.

The anti-inflammatory properties of the MV-Ext, MV-Lipos, Lipo, and the positive control (ibuprofen 10 mg/kg) are illustrated in Figure 6. Three hours after injecting 1% carrageenan into the hind paw of mice treated with the liposomes, the paw edema area gradually increased, reaching a peak of 35.48%. Conversely, pre-treatment with both formulations led to a significant, dose-dependent reduction in paw edema (*p* < 0.05). Specifically, MV-Ext reduced edema by 66.13% at 50 mg/kg and 74.19% at 100 mg/kg. Notably, the anti-edematous activity of MV-Lipos was markedly higher than that of MV-Ext achieving a reduction of 75.81% at 50 mg/kg and 87.10% at 100 mg/kg. Throughout the treatment period, the efficacy of MV-Lipos at 100 mg/kg remained comparable to that of ibuprofen.

### 2.6. In Silico Analysis for Target Prediction of M. vulgare’s Natural Compounds

Natural compounds derived from *Marrubium vulgare* L., exhibited significant potential inhibitory interactions with the active sites of key inflammatory enzymes, notably cyclooxygenases (COX) and lipoxygenases (LOX), in comparison to standard reference drugs, as revealed by molecular docking analyses (Table 5). Apigenin, catechin, kaempferol-3-O-glucuronoside, quercetin-3-O-glucoside, and resveratrol demonstrated favorable binding affinities toward the active site of cyclooxygenase. Quercetin-3-O-glucoside and resveratrol presented the strongest predicted inhibitory activity, with Glide G-scores of −8.980 and −8.807 kcal/mol, respectively, indicating their potential role as effective COX inhibitors in analgesic therapies (Table 5).

In the context of anti-inflammatory activity, isorhamnetin-3-O-rutinoside, kaempferol-3-O-glucuronoside, procyanidin, and sinapic acid demonstrated stronger inhibitory interactions with lipoxygenase compared to ibuprofen, a standard anti-inflammatory reference drug (Glide G-score: −6.946 kcal/mol) (Table 5). Kaempferol-3-O-glucuronoside and procyanidin were identified as the most active LOX inhibitors, with Glide G-scores of −8.638 and −7.27 kcal/mol, respectively. These results underline the potential of these polyphenols as anti-inflammatory agents targeting both COX and LOX pathways, enhancing their therapeutic value.

**Figure 7:**
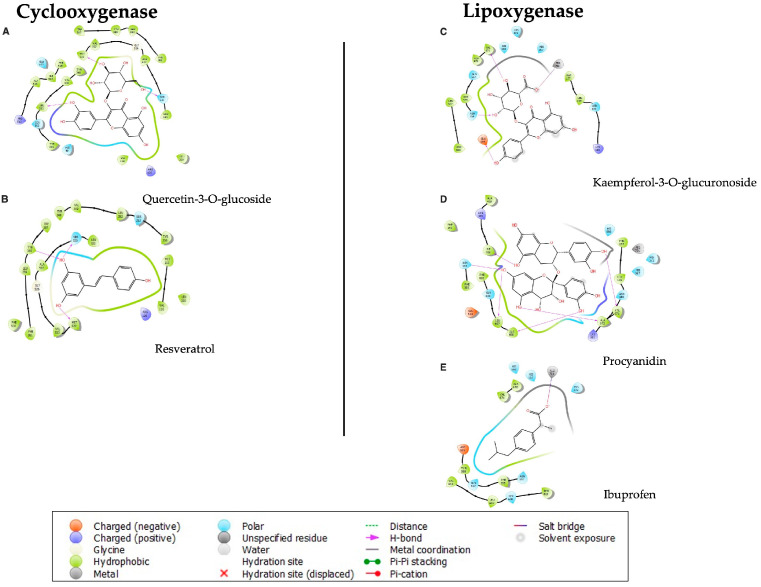
A 2D view of ligand interactions with the active site. (**A**,**B**) Quercetin-3-O-glucoside and resveratrol interactions with the active site of cyclooxygenase COX. (**C**–**E**) Kaempferol-3-O-glucuronoside, procyanidin, and ibuprofen interactions with the active site of lipoxygenase LOX.

**Figure 8:**
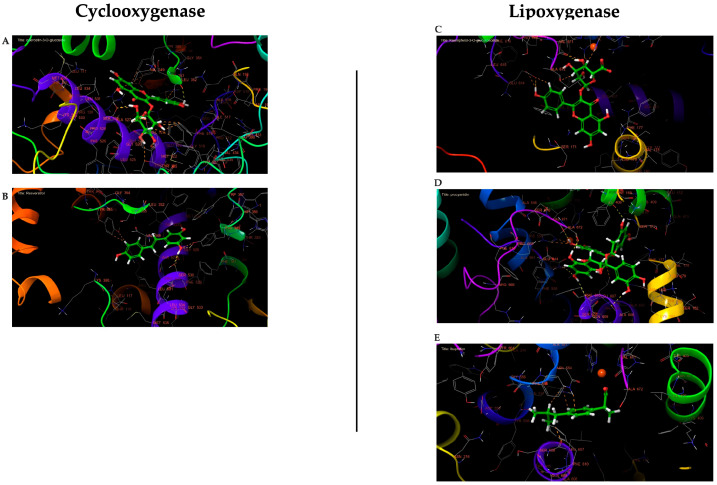
A 3D view of ligand interactions with the active site. (**A**,**B**) Quercetin-3-O-glucoside and Resveratrol interactions with the active site of cyclooxygenase COX. (**C**–**E**) Kaempferol-3-O-glucuronoside, Procyanidin, and Ibuprofen interactions with the active site of lipoxygenase LOX.

Within the active site of COX, quercetin-3-O-glucoside formed three hydrogen bonds with residues MET522, LEU352, and SER530 (Figure 7A and Figure 8A), while resveratrol also established three hydrogen bonds involving TYR385, SER530, and MET522 (Figure 7B and Figure 8B).

Furthermore, in the LOX active site, kaempferol-3-O-glucuronoside formed three hydrogen bonds with residues VAL671, ASN554, and GLU614, along with a salt bridge interaction with the catalytic iron center FE2 701 (Figure 7C and Figure 8C). Procyanidin established six hydrogen bonds involving ILE406, ASN554, LEU607, ALA606, ALA672, and VAL671 (Figure 7D and Figure 8D). In comparison, ibuprofen, the standard anti-inflammatory reference drug, formed only a single salt bridge with FE2 701 (Figure 7E and Figure 8E).

## 3. Discussion

Food and pharmaceutical nanotechnology innovations are advancing rapidly, offering promising applications such as the nano-encapsulation of bioactive compounds for various biological purpose [30]. Among these compounds, polyphenols and flavonoids have been the focus of numerous studies due to their analgesic and anti-inflammatory effects [31].

In traditional Moroccan medicine, *M. vulgare* is frequently used to address inflammation and inflammatory pain [32], primarily due to its polyphenolic compounds. Prior to this study, the analgesic and anti-inflammatory effects of both the polyphenolic extract and its liposome-encapsulated form have not been investigated. In the present work, these nanoformulations were evaluated for their therapeutic activities and sub-acute toxicity profiles. Sample phenolic content measured in MV-Ext was 126 mg GAE/g, consistent with values reported in the literature for this species, which is rich in phenolic compounds and has a content ranging from 48.97 to 195.00 mg GAE/g extract [33,34].

The MV-Lipos were successfully synthesized using a modified thin-film hydration method followed by ultrasonication. The use of high-purity phosphatidylcholine and sodium cholate yielded a stable colloidal suspension. Both MV-Lipos and Lipo exhibited a similar mean size of around 130 nm and a narrow polydispersity index, underscoring the homogeneous and monodisperse nature of the vesicle population. This nanoscale size is particularly suitable for drug delivery, facilitating efficient permeation through the stratum corneum and enhancing interaction with deeper skin layers [35]. Comparable liposomal formulations encapsulating polyphenolic plant extracts, such as *Curcuma longa* or *Camellia sinensis*, have reported size ranges between 100 and 150 nm depending on lipid composition and processing conditions [36,37]. The size obtained in this study aligns well with these findings supporting the potential of the formulation for therapeutic applications. Moreover, the low PDI value observed suggests that the combination of thin-film hydration and optimized ultrasonication effectively yielded well-dispersed nanocarriers. Similar PDI values have been reported for liposomes incorporating polyphenols when precise sonication parameters and phosphatidylcholine-rich lipid matrices are employed [38]. The strong negative zeta potential value indicated substantial electrostatic stabilization. This parameter is critical for predicting colloidal stability, as values beyond ±25 mV generally ensure sufficient electrostatic repulsion to prevent vesicle aggregation [39]. These results are comparable to those reported for liposomes stabilized with bile salts and loaded with anionic polyphenolic compounds such as syringic acid and rosmarinic acid [40]. The inclusion of sodium cholate in the lipid bilayer likely enhanced the surface charge, promoting better dispersion stability. Nanoparticle tracking analysis (NTA) confirmed a high particle concentration of (8.38 ± 0.14) × 10^8^ particles/mL. This concentration is within the optimal range reported for effective topical delivery systems (10^8^–10^9^ particles/mL), ensuring adequate drug load and bioactive compound availability at the target site [41]. The consistency between NTA and DLS data supports the robustness of the preparation method. Furthermore, the lack of size difference between Lipo and MV-Lipos suggests that the incorporation of the natural extract did not compromise vesicle integrity or stability—an encouraging indicator for clinical applications.

The encapsulation efficiency is a critical parameter in evaluating the performance of nanocarriers for nutraceutical delivery. In the present study, MV-Lipos achieved an outstanding encapsulation efficiency of 84%. This represents a significant improvement compared to other lipid-based systems delivering plant polyphenols, which often report lower entrapment rates. However, in this case, the relatively small size and moderate hydrophilicity of syringic acid likely favored its localization near the aqueous interface of the lipid bilayer, facilitating its encapsulation. When compared with similar studies, the obtained encapsulation efficiency aligns well or even outperforms several previously reported results. For instance, Lu Q et al. [42] reported an EE of 60.89% for liposomes loaded with green tea polyphenols using a similar thin-film hydration method. Similarly, Katuwavila et al. [43] achieved EEs ranging from 65% to 70% for caffeic acid encapsulated in phosphatidylcholine-based liposomes. In another study, Camilleri et al. [44] encapsulated olive mill waste in liposomes with an EE of approximately 75%, highlighting how the composition, lipid selection, and sonication parameters influence encapsulation outcomes. The observed EE of 84% thus demonstrates the suitability and efficiency of the developed liposomal system for the delivery of *M. vulgare* polyphenols. High encapsulation not only enhances the chemical stability of the polyphenols by protecting them from oxidation and enzymatic degradation, but also supports sustained release, targeted delivery, and improved bioavailability upon application [42,43,44].

To gain a deeper mechanistic insight into this controlled behavior, the experimental release data were mathematically fitted. According to the Korsmeyer–Peppas classification, values of *n* between 0.45 and 0.89 are associated with non-Fickian diffusion, suggesting that the release process is controlled by a combination of molecular diffusion and structural rearrangement phenomena within the carrier system [45]. The initial burst effect can be attributed to the rapid desorption of polyphenols located near the external surface of the liposomes or weakly associated with the outer phospholipid layers, which are immediately exposed to the release medium. Conversely, the subsequent slower release phase can be associated with the gradual diffusion of polyphenols entrapped within the aqueous core or incorporated into the phospholipid bilayer, together with progressive rearrangement and relaxation processes of the lipid membrane. The predominance of an anomalous transport mechanism indicates that MV-Lipos release is not exclusively controlled by passive diffusion but results from the synergistic contribution of diffusion through the lipid bilayer and dynamic modifications of the vesicular structure [46]. This behaviour is consistent with the intrinsic properties of phospholipid-based nanocarriers, whose membranes exhibit fluidity and permeability changes over time, influencing the release kinetics of encapsulated bioactive molecules [47]. The sustained and controlled release observed for MV-Lipos is particularly advantageous for the development of functional delivery systems, as it may prolong the availability of bioactive compounds while limiting their rapid loss after administration.

Phytochemical analysis of MV-Ext revealed the presence of phenolic compounds such as syringic acid, apigenin, catechin, myricetin, naringin, kaempferol-3-O-glucuronic acid, and rutin, similar to those found in the aerial parts of *M. vulgar*. The results of Aitbaba et al. confirmed that the *M. vulgar* extract from Morocco contained phenolic compounds including kaempferol glucoside, quercetin diglucoside, and methyl gallate [48].

The antioxidant capacity of MV-Lipos was further demonstrated by evaluating its protective effect against lipid peroxidation and oxidative stress in LPS-stimulated RAW264.7 macrophages. LPS induction is well known to trigger massive ROS accumulation, leading to membrane lipid peroxidation, as evidenced by a significant increase in MDA levels. In our study, free MV-Ext showed a moderate reduction in MDA levels, whereas the nano-encapsulated form (MV-Lipos) led to a striking decrease, effectively restoring cellular membrane integrity. This enhanced protective effect was likely due to the liposomal bilayer, which facilitates the internalization of the encapsulated polyphenols into the macrophages, thereby maximizing their scavenging efficiency.

To further elucidate the underlying mechanism, the activities of key endogenous antioxidant enzymes, namely SOD, CAT and GPx, were monitored. These enzymes constitute the primary line of defense against oxidative injury by converting highly reactive superoxide radicals into hydrogen peroxide, which is subsequently detoxified into water and oxygen. Following LPS exposure, a drastic depletion of endogenous SOD, CAT and GPx activities was observed due to overconsumption during chronic oxidative stress. Notably, pre-treatment with MV-Lipos significantly reversed this enzymatic depletion, showing superior efficiency compared to the free extract. This preservation of the intracellular antioxidant defense mechanism underscores the benefit of liposomal encapsulation, which prevented polyphenol degradation and ensured a sustained antioxidant effect within the inflamed microenvironment.

The anti-inflammatory and analgesic properties of these polyphenols significantly reduce paw edema and pain by modulating inflammatory pathways, primarily through interactions with cyclooxygenase enzymes (COX-1 and COX-2), which are responsible for the secretion of prostaglandins implicated in inflammation and pain [49]. Polyphenolic compounds, such as syringic acid, ferulic acid, resveratrol, and rutin, have demonstrated notable anti-inflammatory and analgesic effects in animal models [32,34,50,51]. A study by Yousefi, et al. showed that a 200 mg/kg dose of *M. vulgare* L. methanolic extract exhibited analgesic activity comparable to that of acetylsalicylic acid [49]. In another study, apigenin was found to reduce pro-inflammatory cytokines by lowering plasma levels of IL-1β, IL-6, TNF-α, and PGE2, which are key molecules implicated in edema and pain [52]. In line with these findings, our experimental results showed a considerable reduction in pro-inflammatory cytokines after MV-Lipos administration. This strong action is most likely enabled by the liposomal bilayer, which improves the intracellular distribution and stability of the encapsulated polyphenols. Although MV-Lipos have been shown to reduce oxidative stress and cytokine release, the study did not directly investigate key intracellular pathways like NF-kB or MAPK. Therefore, further biochemical studies are required to clearly establish the precise molecular pathways involved.

Nanoencapsulation through liposomal processes can enhance the bioavailability and efficacy of polyphenols by improving their absorption and allowing for prolonged release in the gastrointestinal tract [53]. Additionally, the mucoadhesive properties of nanoencapsulation promote gastrointestinal retention and targeted release, resulting in more potent effects compared to non-encapsulated compounds [13]. Our nanoencapsulation of polyphenols from *M. vulgare* also demonstrated these effects, showing significant anti-inflammatory and analgesic impacts compared to the control group.

In this sub-acute toxicity study, the oral administration of MV-Ext and MV-Lipos at the tested doses, induced no marked changes in animal behavior, confirming the absence of toxic symptoms. Moreover, body weight analysis showed no significant weight loss, which ruled out undesirable side effects [54]. Interestingly, despite the steady overall body weight progression, a significant reduction in relative liver weight was observed in mice treated with MV-Ext and Lipo. This alteration suggests a potential sub-clinical hepatic stress or metabolic adaptation triggered by the unencapsulated phytocomponents. Remarkably, this phenomenon was completely prevented when the extract was encapsulated within the lipid bilayers, as animals treated with MV-Lipos maintained normal liver weights comparable to the control group. This protective effect strongly indicates that the liposomal carrier successfully modulates the tissue distribution of the extract, minimizing direct hepatic burden and highlighting the superior biocompatibility of our nanoformulation. Regarding biochemical markers, liver enzyme levels (AST and ALT) in all treated group remained completely within normal physiological ranges and showed no statistically significant deviations compared to the healthy control group. This lack of enzymatic elevation provides robust evidence of the safety of the treatments, indicating the hepatoprotective effect of the extract. This was supported by liver histopathological examination, which showed no structural alterations [50]. Similarly, no significant changes in creatinine and urea levels were observed at either tested dose compared to the control group. Since creatinine is a reliable biomarker of renal function, increased levels would suggest damage to functional nephrons. Consequently, the results of this study indicated that neither the Lipo nor the MV-Ext nor the MV-Lipos impaired renal function. These findings suggested that the kidney function of animals treated with the polyphenolic fraction remained intact, as confirmed by histological analysis showing no lesions, consistent with observations from previous studies [51,55].

The docking study highlighted that *M. vulgare* polyphenols, particularly quercetin-3-O-glucoside and resveratrol, have strong potential as natural COX inhibitors with high binding affinities, suggesting a possible mechanism contributing to analgesic therapies. Additionally, kaempferol-3-O-glucuronoside and procyanidin demonstrated significant predicted in silico inhibition of LOX, emphasizing their anti-inflammatory properties. The virtual screening through molecular docking, a widely used in silico method in structure-based drug development, allows for predicting binding orientation and assessing the binding energy of small molecules within the target binding pocket. This approach representing a theoretical framework that requires future direct enzymatic validation.

The molecular docking results, presented in Table 5, indicated that these polyphenols exhibit high binding affinities for COX-2 and 5-LOX, with several candidates showing stronger in silico interaction with LOX than the standard drug ibuprofen, suggesting that these proteins may play a central role in their anti-inflammatory mechanism. These findings provide speculative structural support to the in vivo anti-inflammatory results obtained in this study, indicating that these polyphenols could effectively reduce pain and inflammation potentially by inhibiting key enzymes involved in the prostaglandin and leukotriene pathways. According to the docking results and previous literature, the anti-inflammatory efficacy of *Marrubium vulgare* extract may be mediated through its interaction with leukotriene receptors and cyclooxygenases [56].

Overall, the molecular docking analysis provides computational support for the possible interaction of several *M. vulgare* polyphenols with inflammatory targets. Nevertheless, these findings should be regarded as hypothesis-generating and require further biochemical and pharmacological studies to experimentally validate the proposed molecular interactions and mechanisms of action.

## 4. Materials and Methods

### 4.1. Plant Material

*Marrubium vulgare* L., was collected in April 2021 from the Taounate region (34°37′02.1″ N, 004°51′17.5″ W), Morocco. Professor Amina Bari, a member of the Biology Department at the Faculty of Science Fez, Morocco, conducted the botanical identification, cataloguing the sample with the specimen code number BPRN.15.

### 4.2. Extraction and Characterization of Polyphenolic Compounds from M. vulgare

The aerial parts of the plant were dried in a shaded, well-ventilated environment for one week, then ground into fine particles using a laboratory grinder (IKA M 20 Universal Mill, IKA Werke GmbH & Co, Staufen im Breisgau, Germany). The extraction was performed adhering to the guidelines from [57]. A 10 g sample of the ground defatted powder (rinsed with n-hexane until a clear liquid was obtained) was subjected to ultrasonic-assisted extraction (35 kHz, 40 min, 25 °C) in a methanol/distilled water solution at a ratio of 70:30 (*v*/*v*). The mixture was filtered using Whatman paper and concentrated at 40 °C with a rotary evaporator (IKA RV 10 auto V, IKA Werke GmbH & Co, Staufen im Breisgau, Germany). Then, the extract was diluted with distilled water, and partitioned three times with n-butanol at a 50:50 (*v*/*v*) ratio for 3 h. The final MV-Ext was stored at 4 °C until use.

#### 4.2.1. Total Polyphenol Quantification (TPC)

The total polyphenol content of MV-Ext was calculated using the Folin–Ciocalteu colorimetric method as described by Conte et al. [58]. Briefly, 0.1 mL aliquot of the extract was combined with 0.5 mL Folin–Ciocalteu (Sigma-Aldrich, St. Louis, MO, USA; Cat. No. F9252) diluted 10-fold with distilled water, followed by the addition of 1.5 mL of a 20% sodium carbonate (Na_2_CO_3_) solution. After 2 h incubation period in the dark at room temperature, the absorbance of the resulting solution was measured at 765 nm using a microplate reader, Cytation 5 Cell Imaging Multimode Reader (BioTek, Bad Friedrichshall, Germany). The TPC was expressed as milligrams of gallic acid equivalents per gram of dry weight of the extract (mg of GAE/g DW). Gallic acid (Sigma-Aldrich, St. Louis, MO, USA; Cat. No. G7384) at different concentrations (0.01–0.8 mg/mL) was used as a standard to create a calibration curve. All measurements were performed in triplicate, and the results were averaged for each extract.

#### 4.2.2. Phytochemical Profiling by LC-MS/MS

MV-Ext was analyzed using ultra-high-performance liquid chromatography (UHPLC) coupled with tandem mass spectrometry (MS/MS). The key mass spectrometric parameters were optimized as follows: nebulizing gas flow rate of 3 L/min, heating gas flow rate of 10 L/min, interface temperature of 300 °C, desolvation line (DL) temperature of 250 °C, heat block temperature of 400 °C, and drying gas flow rate of 10 L/min. Chromatographic separation was achieved on a Kinetex C18 column (2.6 µm, 100 Å, 100 × 4.6 mm; Phenomenex Inc., Torrance, CA, USA) using a mobile phase composed of acetonitrile and water (5:95, *v*/*v*) with 0.01% formic acid. Phenolic compounds were identified based on their precursor ion masses (*m*/*z*) using a reference database. Before injection, the extract was diluted with the mobile phase at a 1:5 (*v*/*v*) ratio [59].

### 4.3. Formulation and Characterization of Liposomes Encapsulating MV Polyphenols (MV-Lipos)

Liposomes were formulated using a modified thin-film hydration method with ultrasonication [60]. Briefly, 100 mg of MV-Ext and 2 g of Lipoid S100 (Lipoid GmbH, Ludwigshafen, Germany) were dissolved in 10 mL of organic solvent (1:4 ethanol/chloroform). Once completely solubilized, the organic phase was evaporated under reduced pressure using a rotary evaporator to form a thin lipid film on the flask wall. The lipid film was then hydrated with 75 mL of deionized water, yielding a final phospholipid concentration of 33 mM. To reduce vesicle size and enhance encapsulation, the suspension was subjected to magnetic stirring for 30 min at 30 °C, followed by two ultrasonication cycles: 4 min at pulse mode (output 2) with a 20% dwell cycle, 4 min in pulse mode (output 4) at 30% amplitude (Branson Sonifier 450, Branson Ultrasonics, Danbury, CT, USA). To remove unencapsulated compounds and residual solvents, liposomes were centrifuged using Amicon Ultra centrifugal filters (cut-off 3000 Da) at 4000 rcf and 4 °C for 30 min (OHAUS FC5718R, Parsippany, NJ, USA). Empty liposomes were prepared following the same protocol without the addition of the extract and used as a negative control. The resulting formulations were stored at 4 °C.

The size distribution, polydispersity index (PDI), and surface charge (zeta potential) were obtained using Dynamic Light Scattering (DLS, Zetasizer Nano-ZS instrument, Malvern Instruments, Worcestershire, UK) and Nanoparticle Tracking Analysis (NTA, NanoSight Tracking System, Malvern Instruments, Worcestershire, UK) as reported by De Luca et al. [61]. All analyses were performed in triplicate to ensure reproducibility. Fourier transform infrared (FTIR) spectra were performed using a Thermo Fisher FTIR spectrometer equipped with an attenuated total reflectance (ATR) accessory (Nicolet is50, Thermo Fischer Scientific, Milan, Italy) at frequencies of 500–4000 cm^–1^.

### 4.4. Encapsulation Efficiency (EE)

The encapsulation efficiency (EE) of MV-Lipos was determined using ultrafiltration tubes (MWCO = 3 kDa). MV-Ent and total polyphenols (Wtotal) were measured using liquid chromatography–mass spectrometry (LC-MS), and syringic acid was initially selected as a representative marker compound due to its abundance in the *M. vulgare* extract. However, considering the different physicochemical properties of the identified polyphenols, the encapsulation efficiency was also evaluated for two additional representative compounds, namely verbascoside and quercetin. These compounds were selected to represent different polarity profiles within the MV polyphenolic fraction. Verbascoside, a highly polar phenolic glycoside containing multiple hydroxyl groups and sugar residues, was chosen as representative of hydrophilic polyphenols, whereas quercetin, a flavonoid aglycone characterized by a less polar aromatic structure, was selected as representative of compounds with greater affinity for the hydrophobic phospholipid bilayer.

EE was calculated according to the formula below:

$$EE\;\left(\%\right)=\frac{W_{total}-\;W_{free}\;}{W_{total}\;}\;\times 100$$

### 4.5. In Vitro Release Kinetics of MV-Ext-Loaded Liposomes

The in vitro cumulative release of MV-Ext from liposome formulations was measured using a dialysis bag method (MWCO = 3 kDa) under controlled conditions at 37 °C, in simulated gastrointestinal fluid (SGF, 2 g of sodium chloride, 3.2 g of pepsin, and 7 mL of hydrochloric acid per L (*w* = 36%); pH 1.2) or simulated intestinal fluid (SIF, 6.8 g of potassium phosphate, 10 g pancreatin, and 77 mL of 0.2 M sodium hydroxide per L; pH 6.8). Briefly, 1 mL of MV-Lipos was immersed in a 20 mL of fresh SGF, under gentle agitation at 100 rpm. At predetermined time intervals, 1 mL of release medium was taken out and substituted with the same amount of fresh medium to keep perfect sink conditions. The released MV polyphenol concentration was measured using LC-MS. All measurements were performed in triplicate, and results are expressed as mean ± standard deviation.

For MV-Lipos stability, 5 mL of the liposome dispersion was transferred to a dialysis bag with a molecular weight of 12,000 Da and placed in 25 mL of the SGF and incubated in a shaking incubator at 37 °C for 2 h. Afterward, the bag was placed in the simulated intestinal fluid (comprising 1.81 g/L NaOH, 8.09 g/L K_2_HPO_4_, 4.76 g/L pancreatin, and 5.16 g/L bile salt; pH = 7.5) and incubated again for 6 h. The release percentage was determined at 30 min and 60 min intervals in the gastric and intestinal fluids, respectively [61].

### 4.6. In Vitro Cell Studies

#### 4.6.1. Cell Culture and In Vitro Model

The RAW264.7-derived macrophage cell line was purchased from American Type Culture Collection (ATCC, Manassas, VA, USA) and maintained in RPMI 1640 medium containing 10% fetal bovine serum (FBS) and 1% penicillin/streptomycin (all purchased from Euroclone, Milan, Italy). The cells were kept in an incubator maintaining appropriate culture conditions (37 °C, high humidity, gas phase composed of a mixture of 5% CO_2_/95% atmospheric air). All experiments were performed with an 80% confluent monolayer. To mimic an infection/inflammation environment, macrophages (MΦs) were cultured with ultrapure lipopolysaccharide (LPS, 100 ng/mL, Sigma-Aldrich, Milan, Italy, Cat. No. L2880-10MG) for 24 h. A shorter exposure (4 h) was used to investigate effects on mRNA expressions [62].

#### 4.6.2. Cytocompatibility Assays

The cytotoxicity of MV-Ext and MV-Lipos nanoformulations was assessed following Cell Counting Kit-8 (CCK8, Cat. No. 96992-100TESTS-F) and Lactate Dehydrogenase (LDH, Cat. No. MAK529-1KT) release assay according to the manufacturer’s protocol (Sigma-Aldrich, Milan, Italy). Briefly, cells were plated at 1 × 104 cells/well in 96-well plates and incubated for 24 h in the culture medium containing varying concentrations (20, 40, 60, 80, or 100 µg/mL), of either MV-Ext and MV-Lipos. After that time, 10 μL of CCK-8 solution was added to each well, and the absorbance was measured at 450 nm using a microplate reader (Cytation 3 Microplate Reader, BioTek Instruments, Winooski, VT, USA). Cell viability was determined as a percentage compared to the untreated cells. For membrane damage evaluation, cells were treated as previously described, and LDH released in the culture medium was measured at 490 nm. Triton X-100 (Sigma-Aldrich, Milan, Italy, Cat. No. X100-5ML) solutions (1%) and the culture media only were used as positive and negative controls, respectively [63].

#### 4.6.3. Intracellular Oxidative Stress

The malondialdehyde (MDA) concentration, serving as a lipid peroxidation index, was determined using the thiobarbituric acid reactive substance (TBARS) assay, as per the manufacturer’s protocol. Total SOD-like (Cat. No. CS0009-1KT), GPx (Cat. No. MAK437-1KT), and CAT (Cat. No. CAT100-1KT) activities were measured according to the manufacturer’s protocol (Sigma-Aldrich, Milan, Italy). The result of each activity was determined using a standard curve.

#### 4.6.4. Enzyme-Linked Immunosorbent Assay (ELISA)

The concentration of Interleukin-1β (IL-1β, Cat. No. BMS224-2), Interleukin-6 (IL-6, Cat. No. EH2IL6), and tumor necrosis factor (TNF-α, Cat. No. 88-7346-88), were measured by ELISA (ThermoFisher Scientific, Milan, Italy) as per the manufacturer’s protocol in the supernatants of RAW264.7. Concentrations in samples were determined by interpolation from standard curves.

#### 4.6.5. Cytokine/Chemokine mRNA Expression Levels

To determine the effect of MV-Ext or MV-Lipos on inflammatory gene expression, RAW264.7 cells were incubated with different concentrations of the formulation in the presence or absence of lipopolysaccharide (LPS, 100 µg/mL) to induce an inflammatory response. After 24 h of incubation, the total RNA was isolated using TriFast (EuroClone, Milan, Italy, Cat. No. EMR517100), according to the manufacturer’s protocol, and mRNA levels were measured by qRT-PCR amplification as reported by Valentino et al. [64]. Specific primers for *IL-1β*, *IL-6*, *TNF-α*, and *β-Actin* (*ACTB*) are listed in Table 6. Gene expression was quantified by the 2^−ΔΔCt^ method and normalized against *β*-actin, used as the internal reference gene. Results are expressed as fold changes versus control.

### 4.7. In Vivo Therapeutic Effects

#### 4.7.1. Handling and Housing Animals

Swiss albino mice (adult male and female, aged 6 weeks, 25–35 g, ethical approval number: 04/2023/LEABS) were utilized in this study. Animals were divided into 8 groups (n = 5) and housed under controlled conditions (23 ± 2 °C, 12:12 light–dark cycle, and 45–50% humidity) for a one-week acclimatization. The handling and euthanasia of animals were conducted in accordance with the European Community ethical guidelines [65].

**Scheme 2:**
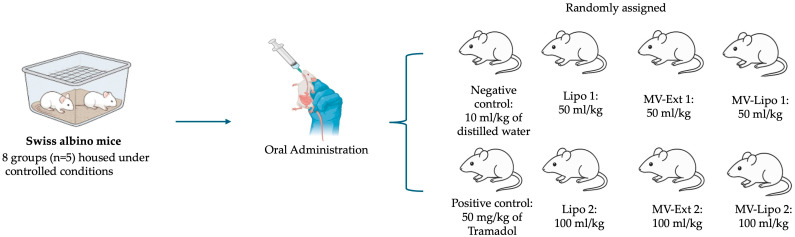
Overview of experimental design, illustrating the division of mice into control and treatment groups for subsequent analysis.

Five animals per study group were randomly assigned to one of eight categories (negative control, positive control, empty Lipo (Lipo1: 50 mg/kg and Lipo 2: 100 mg/kg), MV-Ext (MV-Ext50: 50 mg/kg and MV-Ext100: 100 mg/kg), and MV-Lipos (MV-Lipo50: 50 mg/kg and MV-Lipo100: 100 mg/kg) (Scheme 2). In all models, the first group (negative control) received 10 mL/kg of distilled water, whereas the second group (positive control) administered the standard drug (50 mg/kg of tramadol).

#### 4.7.2. Analgesic Activity In Vivo

The analgesic activity of MV-Ext and MV-Lipos was assessed by counting the number of abdominal contractions in mice following an intraperitoneal injection of 0.7% acetic acid (dose based on body weight) [66]. The samples were administered orally one hour before the injection of acetic acid. After 5 min of latency, the pain-relieving activity was measured by counting the number of writhings which consists of contraction of the abdominal muscle together with stretching of the hind limbs for 30 min. A reduction in the number of writhes as compared to the control group was considered as evidence for analgesic potential activity, and expressed as percent inhibition of writing as follows:

$$\%\;Inhibition\;of\;nociceptive\;response\;capacity=\;\frac{Mean\;no.\;writhers\;\left(control\right)-Mean\;no.\;writhers\;(treated)}{Mean\;no.\;writhers\;\left(control\right)}\;\times 100$$

#### 4.7.3. Anti-Inflammatory Capacity

The anti-inflammatory activity of MV-Ext and MV-Lipos was evaluated using the Paw edema induced by carrageenan (Type IV, essentially pure λ-carrageenan; Sigma-Aldrich, St. Louis, MO, USA; Cat. No. C3889) method, as described by Nalimanana et al. [67]. The acute inflammatory response was induced by a subplantar injection of 50 µL of a 1% (*w*/*v*) carrageenan solution dissolved in sterile saline into the right hind paw of the mice. MV-Ext (50 and 100 mg/kg), MV-Lipos (50 and 100 mg/kg) and ibuprofen (10 mg/kg) were orally administered one hour before the carrageenan injection [68]. The paw volume of mice was measured using a plethysmometer (Ugo Basile 7140, Varese, Italy), before injection and at predetermined intervals (3, 4, 5, and 6 h) post-injection. Finally, the percentage of the swelling in the paw with respect to the mice that took distilled water was calculated using equations below (positive control):

$$\mathrm{Percent}\;\mathrm{inhibition}\;=\;[1\;-\;(\frac{a-x}{b-y})]\;\times \;100$$

where ‘a’: represents the mean edema volume after carrageenan injection. ‘x’ represents the mean edema volume at different time points (3, 4, 5, and 6 h) for the treated groups before the injection. ‘b’ refers to the mean paw volume of control animals after carrageenan injection and ‘y’ is the mean paw volume of control animals before the injection.

#### 4.7.4. Sub-Acute Toxicity

The sub-acute toxicity study was conducted in accordance with the guidelines of the Organization for Economic Co-operation and Development (OECD), Test Guideline 407. Signs of toxicity and body weight were monitored daily, throughout the 4-week study period, both before and after the administration of MV-Ext, MV-Lipos, or Lipo to the rodents. Young mice were divided into eight groups (n = 5 per group; 2 females and 3 males): Group I (negative control) received distilled water, Group II (positive control) received tramadol (50 mg/kg/day), Groups III and IV received Lipo (50 and 100 mg/kg/day, respectively), Groups V and VI received MV-Ext (50 and 100 mg/kg/day, respectively), and Groups VII and VIII received MV-Lipos (50 and 100 mg/kg/day, respectively). At the end of the 28 days, all surviving animals were deeply anesthetized for blood and organ collection [69].

#### 4.7.5. Biochemical Blood Test

After 24 h of fasting, the mice were deeply anesthetized with sodium pentobarbital (30 mg/kg, i.p.) and sacrificed. Blood and organ samples were immediately collected to assess toxicity markers. Blood samples were obtained via cardiac puncture into tubes containing 0.2 mL of heparin (10 U/mL). The tubes were centrifuged at 2000× *g* for 10 min at 4 °C, and the resulting plasma was analyzed for biochemical parameters, including transaminases (AST and ALT), urea, and creatinine.

#### 4.7.6. Relative Weight of Organs

The liver, spleen, and kidneys were excised, washed with ice-cold physiological saline, and weighed using an electronic scale. The experimental groups were compared with the negative control group. Each organ was then preserved in 10% neutral buffered formalin for further analysis. The relative organ weight (ROW) for each mouse was calculated using the following formula [70]:

$$ROW\left(kg\right)=\left(\frac{organ\;weight\left(g\right)}{body\;weight\;\left(g\right)}\right)\;\times \;1000$$

#### 4.7.7. Histopathological Examination

A macroscopic examination of isolated organs (liver, spleen and kidneys) was conducted before fixation to observe any alterations in color or morphology. For the histopathological examination, histological sections were prepared by dehydrating the fixed tissues in a graded series of alcohol, embedding them in paraffin, and slicing them into 5-μm-thick sections using a rotary microtome. The sections were then mounted on slides and stained with hematoxylin and eosin for microscopic observation [71].

### 4.8. In Silico Analysis: Prediction of Potential Targets

#### 4.8.1. Ligand Preparation

Molecular docking simulations were conducted to theoretically evaluate the potential analgesic and anti-inflammatory effects of the identified polyphenol composition of *M. vulgare*. The chemical structures of the main phenolic compounds identified in MV-Ext, along with positive control drugs (tramadol and ibuprofen), were retrieved from the PubChem database in 3D SDF format. The LigPrep module of the Schrödinger software (v11.5) was used to refine these ligands using the OPLS3 force field. Each compound was optimized for its ionization state at a pH of 7.0 ± 2.0, generating up to 32 possible stereoisomers per molecule.

#### 4.8.2. Protein Preparation

The 3D crystal structures of the target proteins were sourced from the Protein Data Bank. COX-2 (PDB ID: 6COX) and LOX (PDB ID: 3V99) were selected as key enzymatic targets to evaluate the potential anti-inflammatory and mediated analgesic properties of the compounds. The receptor structures were prepared and optimized using the Protein Preparation Wizard module within the Schrödinger suite. This process involved structural optimization steps, including the addition of hydrogen atoms, adjustment of bond orders, removal of co-crystallized water molecules, assignment of hydrogen bonds network (at pH 7.0 ± 2.0), and assignment of proper partial atomic charges. Finally, a restrained energy minimization was performed using the OPLS3 force field to resolve steric clashes [72].

#### 4.8.3. Receptor Grid Generation

Receptor grid generations were was performed by defining specific spatial coordinates centered on the binding site of the co-crystallized ligands. For COX-2 (PDB ID: 6COX), the grid center was set at X = 23.375, Y = 23.369, Z = 47.619, while for LOX (PDB ID: 3V99), it was set at X = 18.342, Y = −78.663, Z = −33.950. A cubic grid box with dimensions of 20 × 20 × 20 Å was utilized to encompass the active site cavity. Flexible ligand docking simulations were then performed using the Glide module in Schrödinger-Maestro (v11.5) under the Standard Precision (SP) protocol [73], allowing full conformational flexibility of the ligands.

#### 4.8.4. Glide Standard Precision (SP) Ligand Docking

During the docking process, non-cis/trans amide bonds were penalized to avoid energetically unfavorable geometries. The scaling for Van der Waals interactions for ligand atoms was set to 0.80, and the partial charge cutoff was maintained at 0.15 to modulate steric clashes and electrostatic contributions. The docking results were assessed based on the glide score derived from the energy-minimized poses of the ligands. For each compound, the conformation displaying the lowest Glide Score was chosen as the optimal docking pose [74].

### 4.9. Statistical Analysis

Experimental results are expressed as means ± standard error of the mean (SEM). The data were statistically analyzed using a two-way analysis of variance (ANOVA), followed by Tukey’s multiple comparisons post hoc test with GraphPad Prism 10 software (GraphPad Software, San Diego, CA, USA). The level of significance was established as *p* < 0.05.

## 5. Conclusions

Polyphenols derived from aerial parts of *Marrubium vulgare* L. were formulated into phospholipid nanocapsules to address the challenges commonly associated with natural extracts, including low stability and poor bioavailability, while enhancing quality and their phytochemical quality and biological activity. Both conventional liposomes and polyphenol-loaded liposomes (MV-Lipos) demonstrated nanometric sizes and high encapsulation efficiency for phenolic acids and flavonoids. Pharmacological studies indicated that the incorporated extracts exhibited therapeutic efficacy comparable to standard reference drugs in alleviating inflammation and pain. Furthermore, Lipo, MV-Ext and MV-Lipos show no adverse effects on vital organs in vivo. Consequently, the developed nanoformulations provide a viable method for the oral delivery of the extract, targeting potential applications in the management of acute inflammation and pain-related disorders.

## Figures and Tables

**Table 1:** LC-MS Identification of Compounds in *M. vulgare* Polyphenol Extract. The concentration of each compound was determined by LC-MS analysis and expressed as mg/100 g of *M. vulgare* extract. The relative abundance (%) was calculated as the ratio between the concentration of each identified polyphenol and the total concentration of quantified polyphenolic compounds. Compounds detected below the limit of quantification are reported as <0.1 mg/100 g and their relative abundance was considered as <0.02%. Retention times were obtained under the chromatographic conditions described in the analytical method.

Compounds	Concentration MV (mg/100 g)	Retention Time (min)	% *w*/*w*
Syringic acid	358.8	1.55	47.16
Verbascoside	238.6	3.35	31.36
Apigenin	3.42	6.80	0.45
Quercetin-3-O-glucoside	<0.1	2.60	<0.02
Resveratrol	25.0	2.20	3.29
Isorhamnetin-3-O-rutinoside	25.04	4.90	3.29
Isorhamnetin-7-O-pentose	<0.1	2.90	<0.02
Luteolin-7-O-glucoside	1.31	2.70	0.17
Kaempferol-3-O-glucuronic acid	1.58	3.95	0.21
Sinapic acid	1.82	3.00	0.24
Ferulic acid	1.26	3.50	0.17
Catechin/Epicatechin	1.61	2.80	0.21
Procyanidin	10.61	6.10	1.39
Myricetin	7.92	11.50	1.04
Naringin	4.37	6.80	0.57
Rutin	79.25	6.40	10.42

**Table 2:** Impact of MV-Lipos, MV-Ext and Lipo on weekly body weight gain in groups of mice (n = 5). Values obtained as mean ± SEM.

Parameter	Control	Lipo 50 mg/kg	Lipo 100 mg/kg	MV-Ext 50 mg/kg	MV-Ext 100 mg/kg	MV-Lipos 50 mg/kg	MV-Lipos 100 mg/kg
Initial weight (g)	29.80 ± 0.489	29.20 ± 0.374	30 ± 0.707	31.20 ± 0.583	29.60 ± 0.509	29.80 ± 0.489	30 ± 0.447
One week (g)	36	31.40 ± 0.509	31.30 ± 0.538	32.80 ± 0.200	31.60 ± 0.678	32.00 ± 0.574	32.20 ± 0.374
Two weeks (g)	33.60 ± 0.244	32.20 ± 0.463	33.20 ± 0.374	34.80 ± 0.374	33.40 ± 0.400	34.10 ± 0.556	34 ± 0.273
Three weeks (g)	35.00 ± 0.316	34 ± 0.316	35.40 ± 0.244	36.10 ± 0.640	35.20 ± 0.374	36 ± 0.547	35.90 ± 0.244
Final weight (g)	37.20 ± 0.489	36.60 ± 0.244	36.80 ± 0.374	37.70 ± 0.463	37 ± 0.316	37.20 ± 0.374	37.20 ± 0.374
Body weight (g)	7.400 ± 0.400	7 ± 0.316	6.600 ± 0.509	6.500 ± 0.158	7.400 ± 0.244	7.400 ± 0.509	7.400 ± 0.583

**Table 3:** Relative organ weights of mice orally treated with MV-Lipos, MV-Ext and Lipo. Values are expressed as mean ± SEM (n = 5).

Organ (ROW)	Control	Lipo 50 mg/kg	Lipo 100 mg/kg	MV-Ext 50 mg/kg	MV-Ext 100 mg/kg	MV-Lipos 50 mg/kg	MV-Lipos 100 mg/kg
Liver	7.231 ± 0.166	6.108 ± 0.226 ^ns^	6.156 ± 0.224 ^ns^	6.180 ± 0.513 ^ns^	5.692 ± 0.119	6.924 ± 0.327 ^ns^	7.080 ± 0.280 ^ns^
Kidney	1.284 ± 0.050	1.269 ± 0.050 ^ns^	1.173 ± 0.067 ^ns^	1.020 ± 0.073 ^ns^	0.644 ± 0.058 ^ns^	1.203 ± 0.077 ^ns^	1.404 ± 0.123 ^ns^
Spleen	0.608 ± 0.072	0.624 ± 0.045 ^ns^	0.627 ± 0.44 ^ns^	0.586 ± 0.051 ^ns^	0.6120 ± 0.042 ^ns^	0.633 ± 0.064 ^ns^	0.683 ± 0.603 ^ns^

^ns^: No significant difference compared to the negative control.

**Table 4:** Effects of free and incorporated polyphenolic extracts on liver (aspartate transaminase (AST), alanine aminotransferase (ALT)) and kidney (creatinine (CREA) and urea) quantification during sub-acute toxicity treatment.

Parameter	Control	Lipo 50 mg/kg	Lipo 100 mg/kg	MV-Ext 50 mg/kg	MV-Ext 100 mg/kg	MV-Lipos 50 mg/kg	MV-Lipos 100 mg/kg
AST (U/L)	121.2 ± 1.319	110.6 ± 3.043 ^ns^	111.6 ± 3.868 ^ns^	113 ± 0.748 ^ns^	115.0 ± 0.070 ^ns^	107.4 ± 3.234 ^ns^	108.4 ± 2.619 ^ns^
ALT (U/L)	54 ± 0.447 ^ns^	51 ± 0.447 ^ns^	51.40 ± 0.244 ^ns^	51.40 ± 0.509 ^ns^	53 ± 0.547 ^ns^	52 ± 0.632 ^ns^	52.40 ± 0.244 ^ns^
CREA (mg/L)	3.174 ± 0.021	3.154 ± 0.012 ^ns^	3.050 ± 0.037 ^ns^	3.118 ± 0.060 ^ns^	3.024 ± 0.066 ^ns^	3.036 ± 0.014 ^ns^	3.108 ± 0.032 ^ns^
Urea (g/L)	0.336 ± 0.019	0.296 ± 0.009 ^ns^	0.308 ± 0.008 ^ns^	0.302 ± 0.008 ^ns^	0.314 ± 0.008 ^ns^	0.292 ± 0.013 ^ns^	0.306 ± 0.005 ^ns^

^ns^: No significant difference compared to the negative control.

**Table 5:** Docking results for ligands in different receptors.

	Glide G-Score (kcal/mol)	
	Cyclooxygenase (PDB: 6COX)	Lipooxygenase (PDB: 3V99)
Apigenin	−8.223	−6.693
Catechin	−7.193	−5.681
Ferulic acid	−5.819	−6.555
Isorhamnetin-3-O-rutinoside	-	−6.993
Kaempferol-3-O-glucuronoside	−8.218	−8.638
Luteolin 7-O-glucoside	-	−5.36
Myricetin	−6.686	−5.345
Naringin	-	−6.148
Procyanidin	-	−7.27
Quercetin-3-O-glucoside	−8.98	−5.271
Resveratrol	−8.807	−5.471
Rutin	-	−6.548
Sinapic acid	−6.633	−7.065
Syringic acid	−6.371	−6.333
Verbascoside	-	−6.84
Ibuprofen	-	−6.946

**Table 6:** Primer sequences and accession numbers of target genes used for qRT-PCR amplification. IL-1β, IL-6, and TNF-α were analyzed as inflammatory markers, while ACTB was used as the internal reference gene.

Gene Accession	Number	Forward (5′-3′)	Reverse (5′-3′)
*IL-1β*	NM_000576.3	CGCCTTCGGTCCAGTTGCC	GCCAGTGCCTCTTTGCTGCTTT
*IL-6*	NM_000600.5	TCCAGCTACGAATCTCCGAC	GCATCTTCCTCAGCTTGTCC
*TNF-α*	NM_000594.4	AACATCCAACCTTCCCAAACGC	TGGTCTCCAGATTCCAGATGTCAGG
*ACTB*	NM_001101.5	ACTCTTCCAGCCTTCCTTCC	CGTACAGGTCTTTGCGGATG

## Data Availability

All data generated or analyzed during this study are included in this published article.

## Abbreviations

The following abbreviations are used in this manuscript:

LC-MS/LC-MS/MS	Liquid Chromatography–Mass Spectrometry/Tandem Mass Spectrometry
UHPLC	Ultra-High-Performance Liquid Chromatography
DLS	Dynamic Light Scattering
NTA	Nanoparticle Tracking Analysis
PDI	Polydispersity Index
FTIR	Fourier Transform Infrared Spectroscopy
ATR	Attenuated Total Reflectance
PDB	Protein Data Bank
SDF	Structure Data File
SP	Standard Precision
GAE	Gallic Acid Equivalent/Equivalenti di Acido Gallico
TPC	Total Polyphenol Content
EE/EE%	Encapsulation Efficiency
MV-Ext	*Marrubium vulgare* Crude Extract
MV-Lipos	*Marrubium vulgare* Polyphenol-Loaded Liposomes
IBD	Inflammatory bowel disease
LPS	Lipopolysaccharide
PMA	Phorbol-12-myristate 13-acetate
MΦs	Macrophages
ROS	Reactive Oxygen Species
MDA	Malondialdehyde
TBARS	Thiobarbituric Acid Reactive Substance
SOD	Superoxide Dismutase
CAT	Catalase
GPx	Glutathione Peroxidase
GSH	Reduced Glutathione
TNF-α	Tumor Necrosis Factor-alpha
IL-1β	Interleukin-1 beta
IL-6	Interleukin-6
IL-18	Interleukin-18
NO	Nitric Oxide
PGE_2_	Prostaglandin E_2_
MAPK	Mitogen-Activated Protein Kinase
AP-1	Activator Protein 1
NF-κB	Nuclear Factor kappa-light-chain-enhancer of activated B cells
COX/COX-1/COX-2	Cyclooxygenase
LOX/5-LOX	Lipoxygenase/5-Lipoxygenase
ROW	Relative Organ Weight
AST	Aspartate Aminotransferase
ALT	Alanine Aminotransferase
CREA	Creatinine
SGF	Simulated Gastrointestinal Fluid
SIF	Simulated Intestinal Fluid
TN/TP	Negative Control/Positive Control
SEM	Standard Error of the Mean
SD	Standard Deviation
ANOVA	Analysis of Variance
OECD	Organisation for Economic Co-operation and Development

## References

[1] Mohaddab M., El Goumi Y., Gallo M., Montesano D., Zengin G., Bouyahya A., Fakiri M. (2022). Biotechnology and In Vitro Culture as an Alternative System for Secondary Metabolite Production. Molecules.

[2] Gavarić A., Vladić J., Ambrus R., Jokić S., Szabó-Révész P., Tomić M., Blažić M., Vidović S. (2019). Spray Drying of a Subcritical Extract Using *Marrubium vulgare* as a Method of Choice for Obtaining High Quality Powder. Pharmaceutics.

[3] Marković M., Pljevljakušić D., Matejić J., Rakonjac L., Nikolić B., Djokić M., Jovanović V.S. (2023). Ethnobotanical Investigation of Plants Used for Respiratory Tract Infections in Pirot District (Southeastern Serbia). J. Herb. Med..

[4] Albuquerque U., Patil U., Máthé Á. (2018). Medicinal and Aromatic Plants of South America Brazil.

[5] Amri B., Martino E., Vitulo F., Corana F., Ben-Kaâb L.B., Rui M., Rossi D., Mori M., Rossi S., Collina S. (2017). *Marrubium vulgare* L. Leave Extract: Phytochemical Composition, Antoxidant and Wound Healing Properties. Molecules.

[6] Elberry A.A., Harraz F.M., Ghareib S.A., Nagy A.A., Gabr S.A., Suliaman M.I., Abdel-Sattar E. (2010). Antihepatotoxic effect of *Marrubium vulgare* and *Withania somnifera* extracts on carbon tetrachloride-induced hepatotoxicity in rats. J. Basic Clin. Pharm..

[7] Zarai Z., Kadri A., Ben Chobba I., Ben Mansour R., Bekir A., Mejdoub H., Gharsallah N. (2011). The in-vitro evaluation of antibacterial, antifungal and cytotoxic properties of *Marrubium vulgare* L. essential oil grown in Tunisia. Lipids Health Dis..

[8] Gavarić A., Vladić J., Vujetić J., Radnović D., Volarić A., Živković J., Šavikin K., Vidović S. (2022). The Application of Ultrasonic Waves and Microwaves to Improve Antihyperglycaemic and Antimicrobial Activities of Marrubium vulgare Extracts. Antibiotics.

[9] Abbas M., Saeed F., Anjum F.M., Afzaal M., Tufail T., Bashir M.S., Ishtiaq A., Hussain S., Suleria H.A.R. (2017). Natural polyphenols: An overview. Int. J. Food Prop..

[10] Ansari M.Y., Ahmad N., Haqqi T.M. (2020). Oxidative stress and inflammation in osteoarthritis pathogenesis: Role of polyphenols. Biomed. Pharmacother..

[11] Aguirre A., Borneo R., Watson R.R. (2019). Chapter 4—Improving Bioavailability of Polyphenols Using Nanodelivery Systems Based on Food Polymers. Polyphenols in Plants.

[12] Li J., Li Z., Gao Y., Liu S., Li K., Wang S., Gao L., Shi M., Liu Z., Han Z. (2021). Effect of a Drug Delivery System Made of Quercetin Formulated into PEGylation Liposomes on Cervical Carcinoma In Vitro and In Vivo. J. Nanomater..

[13] Yang B., Dong Y., Wang F., Zhang Y. (2020). Nanoformulations to Enhance the Bioavailability and Physiological Functions of Polyphenols. Molecules.

[14] Păvăloiu R.-D., Sha’at F., Neagu G., Deaconu M., Bubueanu C., Albulescu A., Sha’at M., Hlevca C. (2021). Encapsulation of Polyphenols from *Lycium barbarum* Leaves into Liposomes as a Strategy to Improve Their Delivery. Nanomaterials.

[15] Guimarães D., Cavaco-Paulo A., Nogueira E. (2021). Design of liposomes as drug delivery system for therapeutic applications. Int. J. Pharm..

[16] Gonzalez Gomez A., Hosseinidoust Z. (2020). Liposomes for Antibiotic Encapsulation and Delivery. ACS Infect. Dis..

[17] Salari N., Rasoulpoor S., Valipour E., Mansouri K., Bartina Y., Dokaneheifard S., Mohammadi M., Abam F. (2022). Liposomes, new carriers for delivery of genes and anticancer drugs: A systematic review. Anticancer Drugs.

[18] Liu J., Yang Y., Liu X., Widjaya A.S., Jiang B., Jiang Y. (2021). Macrophage-biomimetic anti-inflammatory liposomes for homing and treating of aortic dissection. J. Control. Release.

[19] Ambati S., Ellis E.C., Pham T., Lewis Z.A., Lin X., Meagher R.B. (2021). Antifungal Liposomes Directed by Dectin-2 Offer a Promising Therapeutic Option for Pulmonary Aspergillosis. mBio.

[20] Allen T.M., Cullis P.R. (2013). Liposomal drug delivery systems: From concept to clinical applications. Adv. Drug Deliv. Rev..

[21] Dizaj S.M., Alipour M., Abdolahinia E.D., Ahmadian E., Eftekhari A., Forouhandeh H., Saadat Y.R., Sharifi S., Vahed S.Z. (2022). Curcumin nanoformulations: Beneficial nanomedicine against cancer. Phytother. Res..

[22] Chopra H., Bibi S., Islam F., Ahmad S.U., Olawale O.A., Alhumaydhi F.A., Marzouki R., Baig A.A., Bin Emran T. (2022). Emerging Trends in the Delivery of Resveratrol by Nanostructures: Applications of Nanotechnology in Life Sciences. J. Nanomater..

[23] Dejeu I.L., Vicaș L.G., Jurca T., Teușdea A.C., Mureșan M.E., Fritea L., Svera P., Gabor G.A., Dejeu G.E., Maghiar O.A. (2021). Liposomes with Caffeic Acid: Morphological and Structural Characterisation, Their Properties and Stability in Time. Processes.

[24] Abu Lila A.S., Ishida T. (2017). Liposomal Delivery Systems: Design Optimization and Current Applications. Biol. Pharm. Bull..

[25] van Alem C.M.A., Metselaar J.M., van Kooten C., Rotmans J.I. (2021). Recent Advances in Liposomal-Based Anti-Inflammatory Therapy. Pharmaceutics.

[26] Lu W.L., Qi X.R. (2019). Liposome-Based Drug Delivery Systems.

[27] Canton M., Sánchez-Rodríguez R., Spera I., Venegas F.C., Favia M., Viola A., Castegna A. (2021). Reactive Oxygen Species in Macrophages: Sources and Targets. Front. Immunol..

[28] Manoharan R.R., Prasad A., Pospíšil P., Kzhyshkowska J. (2024). ROS signaling in innate immunity via oxidative protein modifications. Front. Immunol..

[29] Slighoua M., Chebaibi M., Mahdi I., Amrati F.E.-Z., Conte R., Cordero M.A.W., Alotaibi A., Saghrouchni H., Agour A., Zair T. (2022). The LC-MS/MS Identification and Analgesic and Wound Healing Activities of *Lavandula officinalis* Chaix: In Vivo and In Silico Approaches. Plants.

[30] Faridi Esfanjani A., Jafari S.M. (2016). Biopolymer nano-particles and natural nano-carriers for nano-encapsulation of phenolic compounds. Colloids Surf. B Biointerfaces.

[31] Al-Khayri J.M., Sahana G.R., Nagella P., Joseph B.V., Alessa F.M., Al-Mssallem M.Q. (2022). Flavonoids as Potential Anti-Inflammatory Molecules: A Review. Molecules.

[32] Lefrioui Y., Chebaibi M., Bichara M.D., Mssillou I., Bekkari H., Giesy J.P., Bousta D. (2024). Ethnobotanical survey of medicinal plants used in north-central Morocco as natural analgesic and anti-inflammatory agents. Sci. Afr..

[33] Yousefi K., Hamedeyazdan S., Torbati M., Fathiazad F. (2016). Chromatographic Fingerprint Analysis of Marrubiin in *Marrubium vulgare* L. via HPTLC Technique. Adv. Pharm. Bull..

[34] Aćimović M., Jeremić K., Salaj N., Gavarić N., Kiprovski B., Sikora V., Zeremski T. (2020). *Marrubium vulgare* L.: A Phytochemical and Pharmacological Overview. Molecules.

[35] Honeywell-Nguyen P., Bouwstra J., Honeywell-Nguyen P.L., Bouwstra J.A. (2003). The in vitro transport of pergolide from surfactant-based elastic vesicles through human skin: A suggested mechanism of action. J. Control. Release.

[36] Manca M.L., Cencetti C., Matricardi P., Castangia I., Zaru M., Sales O.D., Nacher A., Valenti D., Maccioni A.M., Fadda A.M. (2016). Glycerosomes: Use of hydrogenated soy phosphatidylcholine mixture and its effect on vesicle features and diclofenac skin penetration. Int. J. Pharm..

[37] Danaei M., Dehghankhold M., Ataei S., Hasanzadeh Davarani F., Javanmard R., Dokhani A., Khorasani S., Mozafari M.R. (2018). Impact of Particle Size and Polydispersity Index on the Clinical Applications of Lipidic Nanocarrier Systems. Pharmaceutics.

[38] Dawoud M.H.S., Yassin G.E., Ghorab D.M., Morsi N.M. (2019). Insulin Mucoadhesive Liposomal Gel for Wound Healing: A Formulation with Sustained Release and Extended Stability Using Quality by Design Approach. AAPS PharmSciTech.

[39] Mozafari M.R., Johnson C., Hatziantoniou S., Demetzos C. (2008). Nanoliposomes and their applications in food nanotechnology. J. Liposome Res..

[40] Ranjbar S., Emamjomeh A., Sharifi F., Zarepour A., Aghaabbasi K., Dehshahri A., Sepahvand A.M., Zarrabi A., Beyzaei H., Zahedi M.M. (2023). Lipid-Based Delivery Systems for Flavonoids and Flavonolignans: Liposomes, Nanoemulsions, and Solid Lipid Nanoparticles. Pharmaceutics.

[41] Dragicevic N., Maibach H. (2017). Percutaneous Penetration Enhancers Drug Penetration Into/Through the Skin. Methodology and General Considerations.

[42] Lu Q., Li D.C., Jiang J.-G. (2011). Preparation of a Tea Polyphenol Nanoliposome System and Its Physicochemical Properties. J. Agric. Food Chem..

[43] Katuwavila Nuwanthi P., Perera A.D.L., Chandani, Karunaratne V., Amaratunga, Gehan A.J., Karunaratne D.N. (2016). Improved Delivery of Caffeic Acid through Liposomal Encapsulation. J. Nanomater..

[44] Camilleri D., Attard K., Lia F. (2025). Formulation and Evaluation of Liposome-Encapsulated Phenolic Compounds from Olive Mill Waste: Insights into Encapsulation Efficiency, Antioxidant, and Cytotoxic Activities. Molecules.

[45] Lakshani N., Wijerathne H.S., Sandaruwan C., Kottegoda N., Karunarathne V. (2023). Release Kinetic Models and Release Mechanisms of Controlled-Release and Slow-Release Fertilizers. ACS Agric. Sci. Technol..

[46] De Sousa Sabino L.B., Copes F., Saulais S., De Brito E.S., Da Silva Júnior I.J., Le T.C., Mateescu M.A., Mantovani D. (2022). Anthocyanins Formulated with Carboxymethyl Starch for Gastric and Intestinal Delivery. Molecules.

[47] Woo Y., Yoon J., Seo Y., Han Y., Yoo H.Y., Sohn H., Lee M., Lee T. (2025). Structure-centered design of lipid-based nanocarriers to overcome biological barriers. Mater. Today Bio.

[48] Aitbaba A., Kabdy H., Fatimzahra A., Azraida H., Abdoussadeq O., Aboufatima R., El Yazouli L., Sokar Z., Garzoli S., Chait A. (2025). Anti-inflammatory and antiarthritic properties of *Marrubium vulgare* aqueous extract in an animal model. Nat. Prod. Res..

[49] Yousefi K., Fathiazad F., Soraya H., Rameshrad M., Maleki-Dizaji N., Garjani A. (2014). Marrubium vulgare L. methanolic extract inhibits inflammatory response and prevents cardiomyocyte fibrosis in isoproterenol-induced acute myocardial infarction in rats. Bioimpacts.

[50] Mssillou I., Agour A., Slighoua M., Chebaibi M., Amrati F.E.-Z., Alshawwa S.Z., Al Kamaly O., El Moussaoui A., Lyoussi B., Derwich E. (2022). Ointment-Based Combination of *Dittrichia viscosa* L. and *Marrubium vulgare* L. Accelerate Burn Wound Healing. Pharmaceuticals.

[51] Parray F., Shafi S., Hussein I.M., Khan I.A., Ali Z., Masoodi M.H., Rehman M.U. (2022). *Marrubium vulgare* L.: Traditional Uses, Phytochemistry, and Pharmacological Profile. Edible Plants in Health and Diseases: Volume II: Phytochemical and Pharmacological Properties Singapore.

[52] Chabane M.A., Tir Touil A., Khelladi B., Meddah B., Mokhtar M. (2020). In Vivo Toxicological and Microbiological Activity of *Marrubium vulgare* L. on *Candida albicans* Isolated from Nosocomial Infections. Pharm. Sci..

[53] Enaru B., Socaci S., Farcas A., Socaciu C., Danciu C., Stanila A., Diaconeasa Z. (2021). Novel Delivery Systems of Polyphenols and Their Potential Health Benefits. Pharmaceuticals.

[54] van Berlo D., Woutersen M., Muller A., Pronk M., Vriend J., Hakkert B. (2022). 10% Body weight (gain) change as criterion for the maximum tolerated dose: A critical analysis. Regul. Toxicol. Pharmacol..

[55] Mssillou I., Agour A., Lefrioui Y., Chebaibi M. (2024). LC-TOFMS analysis, in vitro and in silico antioxidant activity on NADPH oxidase, and toxicity assessment of an extract mixture based on *Marrubium vulgare* L. and *Dittrichia viscosa* L. J. Biol. Biomed. Res..

[56] Emam M., El-Newary S.A., Aati H.Y., Wei B., Seif M., Ibrahim A.Y. (2024). Anti-Alzheimer’s Potency of Rich Phenylethanoid Glycosides Extract from *Marrubium vulgare* L.: In Vitro and In Silico Studies. Pharmaceuticals.

[57] Andersen O.M., Markham K.R. (2005). Flavonoids: Chemistry, Biochemistry and Applications.

[58] Conte R., Sepe F., Margarucci S., Costanzo E., Petillo O., Peluso G., Marcolongo L., Calarco A. (2025). Functional Plant-Based Beverage Fortified with Hazelnut Cuticle Polyphenols: Antioxidant and Phenolic Content Characterization. Molecules.

[59] Slighoua M., Mahdi I., Moussaid F.Z., Al Kamaly O., Amrati F.E.-Z., Conte R., Drioiche A., Saleh A., Housseini A.I., Bari A. (2023). LC-MS/MS and GC/MS Profiling of Petroselinum sativum Hoffm. and Its Topical Application on Burn Wound Healing and Related Analgesic Potential in Rats. Metabolites.

[60] Bochicchio S., Barba A.A., Grassi G., Lamberti G. (2016). Vitamin delivery: Carriers based on nanoliposomes produced via ultrasonic irradiation. LWT-Food Sci. Technol..

[61] De Luca I., Di Cristo F., Conte R., Peluso G., Cerruti P., Calarco A. (2023). In-Situ Thermoresponsive Hydrogel Containing Resveratrol-Loaded Nanoparticles as a Localized Drug Delivery Platform for Dry Eye Disease. Antioxidants.

[62] Tan C., Feng B., Zhang X., Xia W., Xia S. (2016). Biopolymer-coated liposomes by electrostatic adsorption of chitosan (chitosomes) as novel delivery systems for carotenoids. Food Hydrocoll..

[63] Aloi N., Calarco A., Curcuruto G., Di Natale M., Augello G., Carroccio S.C., Cerruti P., Cervello M., Cuttitta A., Colombo P. (2024). Photoaging of polystyrene-based microplastics amplifies inflammatory response in macrophages. Chemosphere.

[64] Valentino A., Conte R., De Luca I., Di Cristo F., Peluso G., Bosetti M., Calarco A. (2022). Thermo-Responsive Gel Containing Hydroxytyrosol-Chitosan Nanoparticles (Hyt@tgel) Counteracts the Increase of Osteoarthritis Biomarkers in Human Chondrocytes. Antioxidants.

[65] Hubrecht R. (2011). Guide for the Care and Use of Laboratory Animals.

[66] Hernández-Pérez M., Rabanal R.M. (2002). Evaluation of the antinflammatory and analgesic activity of *Sideritis canariensis* var. pannosa in mice. J. Ethnopharmacol..

[67] Nalimanana N.R., Tombozara N., Razafindrakoto Z.R., Andrianjara C., Ramanitrahasimbola D. (2022). Anti-inflammatory and analgesic properties, and toxicity of the seed’s ethanol extract of *Calophyllum inophyllum* L. from the eastern region of Madagascar. S. Afr. J. Bot..

[68] GB B.D., Amutha K. (2011). Anti-Inflammatory and Anti-Pyretic Activities of Blumea mollis (D. Don) MERR. Res. J. Pharmacogn. Phytochem..

[69] OECD (2008). Test No. 407: Repeated Dose 28-Day Oral Toxicity Study in Rodents [Internet].

[70] Ramadan A., Soliman G., Mahmoud S.S., Nofal S.M., Abdel-Rahman R.F. (2012). Evaluation of the safety and antioxidant activities of *Crocus sativus* and *Propolis* ethanolic extracts. J. Saudi Chem. Soc..

[71] Amrati F.E.-Z., Elmadbouh O.H.M., Chebaibi M., Soufi B., Conte R., Slighoua M., Saleh A., Al Kamaly O., Drioiche A., Zair T. (2023). Evaluation of the toxicity of Caralluma europaea (C.E) extracts and their effects on apoptosis and chemoresistance in pancreatic cancer cells. J. Biomol. Struct. Dyn..

[72] Amrati F.E.-Z., Chebaibi M., de Azevedo R.G., Conte R., Slighoua M., Mssillou I., Kiokias S., Gomes A.d.F., Pontes G.S., Bousta D. (2023). Phenolic Composition, Wound Healing, Antinociceptive, and Anticancer Effects of Caralluma europaea Extracts. Molecules.

[73] Slighoua M., Amrati F.E.-Z., Chebaibi M., Mahdi I., Al Kamaly O., El Ouahdani K., Drioiche A., Saleh A., Bousta D. (2023). Quercetin and Ferulic Acid Elicit Estrogenic Activities In Vivo and In Silico. Molecules.

[74] Beniaich G., Hafsa O., Maliki I., Bin Jardan Y.A., El Moussaoui A., Chebaibi M., Agour A., Zouirech O., Nafidi H.-A., Khallouki F. (2022). GC-MS Characterization, In Vitro Antioxidant, Antimicrobial, and In Silico NADPH Oxidase Inhibition Studies of Anvillea radiata Essential Oils. Horticulturae.

